# ﻿Comparative analysis of the mitogenomes of two *Corydoras* (Siluriformes, Loricarioidei) with nine known *Corydoras*, and a phylogenetic analysis of Loricarioidei

**DOI:** 10.3897/zookeys.1083.76887

**Published:** 2022-01-24

**Authors:** Cheng-He Sun, Qi Huang, Xiao-Shu Zeng, Sha Li, Xiao-Li Zhang, Ya-Nan Zhang, Jian Liao, Chang-Hu Lu, Bo-Ping Han, Qun Zhang

**Affiliations:** 1 Department of Ecology and Institute of Hydrobiology, Jinan University, Guangzhou 510632, China Jinan University Guangzhou China; 2 College of Biology and the Environment, Nanjing Forestry University, Nanjing 210037, China Nanjing Forestry University Nanjing China; 3 Chinese Sturgeon Research Institute, China Three Gorges Corporation, Yichang 443100, Hubei, China Chinese Sturgeon Research Institute, China Three Gorges Corporation Yichang China; 4 Hubei Key Laboratory of Three Gorges Project for Conservation of Fishes, Yichang 443100, Hubei, China Hubei Key Laboratory of Three Gorges Project for Conservation of Fishes Yichang China

**Keywords:** *Corydorasaeneus*, *Corydoraspaleatus*, genome sequencing, mitochondrial DNA, Phylogenetic tree

## Abstract

*Corydoras* is a speciose catfish genus from South America with widely investigated phylogenetic and evolutionary relationships. The complete mitogenomes of *C.aeneus* and *C.paleatus* were sequenced, assembled, and annotated using next-generation sequencing. The genome arrangements, gene contents, genome structures, base compositions, evolutionary features, codon usage, and tRNA structures of the two mitogenomes were compared and analyzed with nine published mitogenomes of *Corydoras*. Phylogenetic analysis was performed using concatenated nucleotide sequences with 13 protein-coding genes and two rRNAs with 44 mitogenomes of Siluriformes. These results provide information on the mitogenomes of eleven *Corydoras* species and evolutionary relationships within the suborder Loricarioidei, which may be applicable for further phylogenetic and taxonomic studies on Siluriformes and Loricarioidei.

## ﻿Introduction

Fish mitochondrial DNA shares characteristics with other vertebrate mitochondrial DNA (Anderson et al. 1981; Manchado et al. 2007; Xu et al. 2011), e.g., small molecular weight, simple structure, and compact arrangement. It exists in the form of a covalently closed circular supercoil structure and contains heavy and light chains. The genetic material can be replicated, transcribed, and translated independently from the nuclear DNA in the cell. With few exceptions, fish mitochondrial DNA comprises 13 protein-coding genes (PCGs), 22 transfer RNA genes, two ribosomal RNA genes, original region of light-strand replication, and control region (D-loop) (Ojala et al. 1981; Gadaleta et al. 1989; [Bibr B65][Bibr B62]; De Rijk et al. 1995). The mitochondrial DNA mutates rapidly, nearly 10-fold faster than the nuclear DNA, and the fragment length and evolution rate differ for each gene, providing molecular evidence for studying different species (Brown et al. 1979; Pesole et al. 1999). In addition, mitochondrial DNA is highly heterogeneous and harbors the genetic characteristics associated with maternal traits (O’Brien 1971; Michot et al. 1990; Bartlett and Davidson 1991; Meyer 1993; Beheregaray and Sunnucks 2001; Liu et al. 2002; Yoshizawa and Johnson 2003). Hence, mitochondrial DNA can be used to identify fish groups at the molecular level and explore geographic distribution, species origin, and species differentiation (Avise et al. 1987; Kai et al. 2002; Hrbek et al. 2007). As fish are a large group with a complex origin in the vertebrate subphylum, studies on their phylogenetic and evolutionary relationships performed using traditional morphological methods often provide limited information. With advances in biotechnology, complete mitochondrial genome sequences have been determined as a useful tool to study the phylogeny and phylogeography of fish (Bermingham and Avise 1986; Xu et al. 2020).

*Corydoras* Lacépède, 1803, belongs to the order Siluriformes, suborder Loricarioidei, family Callichthyidae. *Corydoras* contains 175 valid species, which makes it the most species-rich genus of the family Callichthyidae (Lima and Britto 2020; Tencatt et al. 2021). The body of these fish is covered with bone plates, and the pectoral and dorsal fins have hard spines that can be used for protection. In addition, *Corydoras* can use the back end of their intestines, which is rich in blood vessels, to obtain oxygen from air taken in at the water surface, enabling survival under environmental stress, such as drought or insufficient dissolved oxygen content in water. *Corydoras* catfish are benthic omnivorous fish (Moreira et al. 2016b, 2017; Liu et al. 2019b, 2019c; Saitoh et al. 2003). Typically, *Corydoras* is active only during feeding, and otherwise hide while resting. *Corydoras* is primarily distributed in South America. Most species of *Corydoras* gather in the middle and lower reaches of the river where the current is relatively gentle, whereas a few live in the upper reaches of the river in rapids (Saitoh et al. 2003; Liu et al. 2019c). *Corydoras* is also valuable as an ornamental fish. Some phylogenetic relationships in *Corydoras* remain unclear. The number of species reported in relevant articles is small, which is not sufficient to reflect the phylogenetic variety of the genus *Corydoras* (Alexandrou et al. 2011; Lujan et al. 2015; Roxo et al. 2019). Therefore, a comprehensive understanding of the relationships between different species of *Corydoras* is essential.

In this study, the complete mitogenomes of two species of *Corydoras* (Bronze corydoras *C.aeneus* Gill, 1858 and peppered corydoras *C.paleatus* Jenyns, 1842) were sequenced, assembled, and annotated. The genome organization, gene contents, repeat sequences, and tRNA structures of the eleven mitogenomes were compared and analyzed in combination with nine published mitogenomes of *Corydoras* (Saitoh et al. 2003; Moreira et al. 2016a, 2017; Liu et al. 2019a, b, c, d; Chen et al. 2020; Lv et al. 2020). Determining the similarities and differences in gene orders, genetic structures, base compositions, evolutionary features, and codon usage can provide molecular insights into the taxonomic and phylogenetic characteristics of the order Siluriformes. Based on these data, and those obtained from the NCBI database, we examined the phylogenetic relationships among species in the suborder Loricarioidei. We also evaluated the mitogenomes of eleven species of *Corydoras* and evolutionary relationships within the suborder Loricarioidei, thereby providing a valuable basis for further evolutionary studies on Siluriformes and Loricarioidei.

## ﻿Materials and methods

### ﻿Sample collection and identification

Single specimens of *C.aeneus* and *C.paleatus* were collected from the temple of Confucius flower and wood fish market, Nanjing city, Jiangsu province, China (32°0'27.1"N, 118°50'11.5"E) in June 2020 and identified based on their morphological characteristics, according to the latest taxonomic classification of fish (Popazoglo and Boeger 2000; Huysentruyt and Adriaens 2005a, b). Their geographic data and specific origins were unknown. All fresh tissues were immediately stored at -80 °C in 95% ethanol until DNA extraction. Total DNA was extracted from the muscle tissue using a TIANamp Marine Animals DNA Kit DP324 (Tiangen Biotech Co., Ltd., Beijing, China) according to the manufacturer’s instructions. DNA integrity and purity were evaluated by 1% agarose gel electrophoresis, and DNA purity was determined with a NanoDrop 2000 (NanoDrop Technologies, Wilmington, DE, USA). DNA concentrations were quantified using a Qubit^R^ 2.0 Fluorometer (Life Technologies, Carlsbad, CA, USA). To ensure the accuracy of morphological identification, COI primers were designed based on the latest DNA barcoding database (NCBI and FishBase) and were amplified, sequenced, and compared. The COI sequences are provided in the Suppl. material 1. The results of the sequence alignment verify the accuracy of the morphological identification.

### ﻿Genome sequencing and assembly

Next-generation sequencing was performed to determine the complete mitogenome sequence of the two species of *Corydoras*. The DNA libraries were sequenced on an Illumina sequencing platform by Novogene Co., Ltd. (Beijing, China). Briefly, the total DNA genome was quantified and fragmented into 250-base pair (bp) fragments using a Covaris M220 ultrasonic crushing system (Woburn, MA, USA) followed by whole-genome shotgun sequencing. According to the manufacturer’s instructions, a library was constructed based on two indices using an Illumina TruSeq DNA PCR-Free HT kit (San Diego, CA, USA). An Illumina Novaseq 6000 platform was used for sequencing of 150 paired-end reads approximately 4 Gb in size. Clean reads were generated as previously described, and the remaining high-quality reads were assembled using SPADES V3.15.2 (Bankevich et al. 2012) (http://cab.spbu.ru/software/spades/) and SOAPDENOVO2 V2.01 (Luo et al. 2012) software. The preliminary assembly results were compared with the NT database, and looped sequences annotated as mitochondrial genomes were screened. CAP3 was used to merge the splicing results from the two software programs, and the assembly results were compared with those of related species using MUMMER v3.23 (Delcher et al. 2003). The mitogenome composition was confirmed, and a complete, high-quality map of the mitochondrial genome was obtained.

### ﻿Genome annotation and analysis

The tRNA genes were verified using tRNASCAN-SE V1.3.1 (Lowe and Eddy 1997) with default settings for the vertebrate mitochondrial genetic code. The software, which integrates multiple analysis tools, can identify 99% of the tRNA genes with a very low number of false positives and predict the secondary structure of tRNAs. Protein-coding regions were re-identified using GLIMMER V3.0 (Ingram et al. 2009), and manual comparisons were performed using the SEQMAN program of LASERGENE V7.1 (Burland 2000) (DNAStar, Inc., Madison, WI, USA) based on the PCGs of nine species of *Corydoras* and translated into putative proteins via GenBank. The non-coding RNAs were verified using RFAM V12.0 (Griffiths-Jones et al. 2003) and INFERNAL V1.1 (Nawrocki and Eddy 2013). The rRNA genes were assumed to extend to the boundaries of flanking genes, similar to the homologous regions of other published mitogenomes of *Corydoras* in GenBank. The MITOS WebServer (http://mitos2.bioinf.uni-leipzig.de/index.py) and MitoFish (Iwasaki et al. 2013) (http://mitofish.aori.u-tokyo.ac.jp/) online tools were used for the final annotation of the entire mitogenome sequence of the two species of *Corydoras*, and the annotated mitogenomes were compared with nine published mitogenomes of *Corydoras*. Base compositions, genetic distances, and relative synonymous codon usage values were determined using MEGA V7.0 (Kumar et al. 1994). A graph comparing the relative synonymous codon usage was drawn using PHYLOSUITE V1.2.2 (Zhang et al. 2020). Strand asymmetry was analyzed using the formula: AT-skew = (A – T)/(A + T). The numbers of non-synonymous (Ka) and synonymous (Ks) substitutions and the ratio of Ka/Ks and nucleotide diversity for the nine species of *Corydoras* were calculated using DNASP 5.1 (Librado and Rozas 2009). The MitoFish (http://mitofish.aori.u-tokyo.ac.jp/) online tool was used to generate circular mitogenome maps.

### ﻿Phylogenetic analysis

Phylogenetic trees for the eleven mitogenomes of *Corydoras* within the family Callichthyidae and Suborder Loricarioidei were constructed by aligning 13 PCGs and two rRNA sequences with those of 42 species of Loricarioidei, 29 species from Loricariidae, and one species from Trichomycteridae (Table 1). The mitogenomes of *Pterocryptiscochinchinensis* (Resende et al. 2016) and *Silurusasotus* (Nakatani et al. 2011) (accession no. NC_027107.1 and NC_015806.1, respectively, suborder Siluroidei) were included as outgroups to root the Loricarioidei tree. All operations were performed in PHYLOSUITE V1.2.2 (Zhang et al. 2020) software package. The nucleotide sequences of 13 PCGs from 44 mitogenomes were aligned in batches with MAFFT V7.313 (Katoh and Standley 2013) (https://mafft.cbrc.jp/alignment/server/) using the codon alignment mode. The results were optimized using MACSE V2.03 (Ranwez et al. 2018). The nucleotide sequences of two rRNAs were aligned using the online tool MAFFT with default settings. Ambiguously aligned regions were removed via GBLOCKS 0.91 b with default settings. The resulting alignments were concatenated into a single dataset with PHYLOSUITE. The best partition schemes and optimal substitution models were selected by MODELFINDER (Kalyaanamoorthy et al. 2017) with the greedy algorithm and Bayesian information criterion (Watanabe 2013). The best substitution models applied to each partition are listed in Suppl. material 1: Table S1. Phylogenetic trees were constructed using two inference methods: maximum likelihood (ML) and Bayesian inference (BI). ML analyses were performed with IQ-TREE V1.6.8 with the models selected for each partition, and 1,000 bootstrap replicates were used to estimate node reliability. Bayesian analyses were performed using MRBAYES V3.2.6 (Huelsenbeck and Ronquist 2001). One million generations of two independent runs were performed with four chains and sampling trees every 100 generations. The initial 25% of trees generated prior to reaching stable log-likelihood values were discarded as burn-in. The remaining trees were used to calculate the Bayesian posterior probabilities. The resulting phylogenetic trees and gene orders were visualized and edited using iTOL (Letunic and Bork 2016).

**Table 1.** Information on 44 Siluriformes species evaluated in the study.

No.	Suborder	Family	Taxa	GenBank accession no.	Length (bp)	Location/Reference
1	Loricarioidei	Callichthyidae	*Corydorasaeneus*	MZ571336	16604	This study
2			*Corydorasagassizii*	MN641875.1	16538	Lv et al. 2020
3			*Corydorasarcuatus*	NC_049096.1	16177	Liu et al. 2019d
4			*Corydorasduplicareus*	NC_049095.1	16632	Liu et al. 2019a
5			*Corydorasnattereri*	KT239008.1	16557	Moreira et al. 2016a
6			*Corydoraspaleatus*	MZ571337	16320	This study
7			*Corydoraspanda*	NC_049097.1	16398	Liu et al. 2019b
8			*Corydorasrabauti*	NC_004698.1	16711	Saitoh et al. 2003
9			*Corydorasschwartzi*	KT239007.1	15671	Moreira et al. 2017
10			*Corydorassterbai*	NC_048967.1	16520	Liu et al. 2019c
11			*Corydorastrilineatus*	NC_049098.1	15359	Chen et al. 2020
12			*Hoplosternumlittorale*	KX087170.1	16262	Parente et al. 2018
13		Loricariidae	*Ancistomussnethlageae*	KX087166.1	16464	Moreira et al. 2017
14			*Ancistruscryptophthalmus*	MF804392.1	16333	Lv et al. 2020
15			*Ancistrusmultispinis*	KT239006.1	16539	Moreira 2018
16			*Ancistrustemminckii*	NC_051963.1	16439	Meng et al. 2021
17			*Aphanotorulusemarginatus*	KT239019.1	16597	Moreira et al. 2017
18			*Baryancistrusxanthellus*	KX087167.1	16167	Moreira et al. 2017
19			*Dekeyseriaamazonica*	KX087168.1	16409	Moreira 2018
20			*Hemipsilichthysnimius*	KT239011.1	16477	Moreira et al. 2017
21			*Hisonotusthayeri*	KX087173.1	16269	Moreira et al. 2017
22			*Hypancistruszebra*	KX611143.1	16202	Magalhães et al. 2017
23			*Hypoptopomaincognitum*	NC_028072.1	16313	Moreira et al. 2016b
24			*Hypostomusaffinis*	KT239013.1	16330	Moreira et al. 2017
25			*Hypostomusancistroides*	NC_052710.1	16422	Rocha-Reis et al. 2020
26			*Hypostomusfrancisci*	NC_045188.1	16916	Pereira et al. 2019
27			*Hypostomusplecostomus*	NC_025584.1	16562	Liu et al. 2016
28			*Kronichthysheylandi*	KT239014.1	16632	Moreira et al. 2017
29			*Loricariacataphracta*	KX087174.1	16831	Moreira et al. 2017
30			*Loricariichthyscastaneus*	KT239015.1	16521	Moreira et al. 2017
31			*Loricariichthysplatymetopon*	KT239018.1	16521	Moreira et al. 2017
32			*Neoplecostomusmicrops*	KX087175.1	16523	Moreira et al. 2017
33			*Otocinclusaffinis*	MT323116.1	16501	Zhang et al. 2021
34			*Pareiorhaphisgarbei*	KX087178.1	16630	Moreira et al. 2017
35			*Parotocinclusmaculicauda*	KX087179.1	16541	Moreira et al. 2017
36			*Peckoltiafurcata*	KX087180.1	16497	Moreira et al. 2017
37			*Pterygoplichthysanisitsi*	KT239003.1	16636	Parente et al. 2017
38			*Pterygoplichthysdisjunctivus*	NC_015747.1	16667	Nakatani et al. 2011
39			*Pterygoplichthyspardalis*	KT239016.1	16822	Moreira et al. 2017
40			*Schizolecisguntheri*	KT239017.1	16611	Moreira et al. 2017
41			*Sturisomatichthyspanamensis*	NC_045877.1	16526	Ren et al. 2019
42		Trichomycteridae	*Trichomycterusareolatus*	AP012026.1	16657	Nakatani et al. 2011
43	Siluroidei	Siluridae	*Pterocryptiscochinchinensis*	NC_027107.1	16826	Resende et al. 2016
44			*Silurusasotus*	NC_015806.1	16593	Nakatani et al. 2011

## ﻿Results and discussion

### ﻿Genome structure and organization

**Figure 1.**
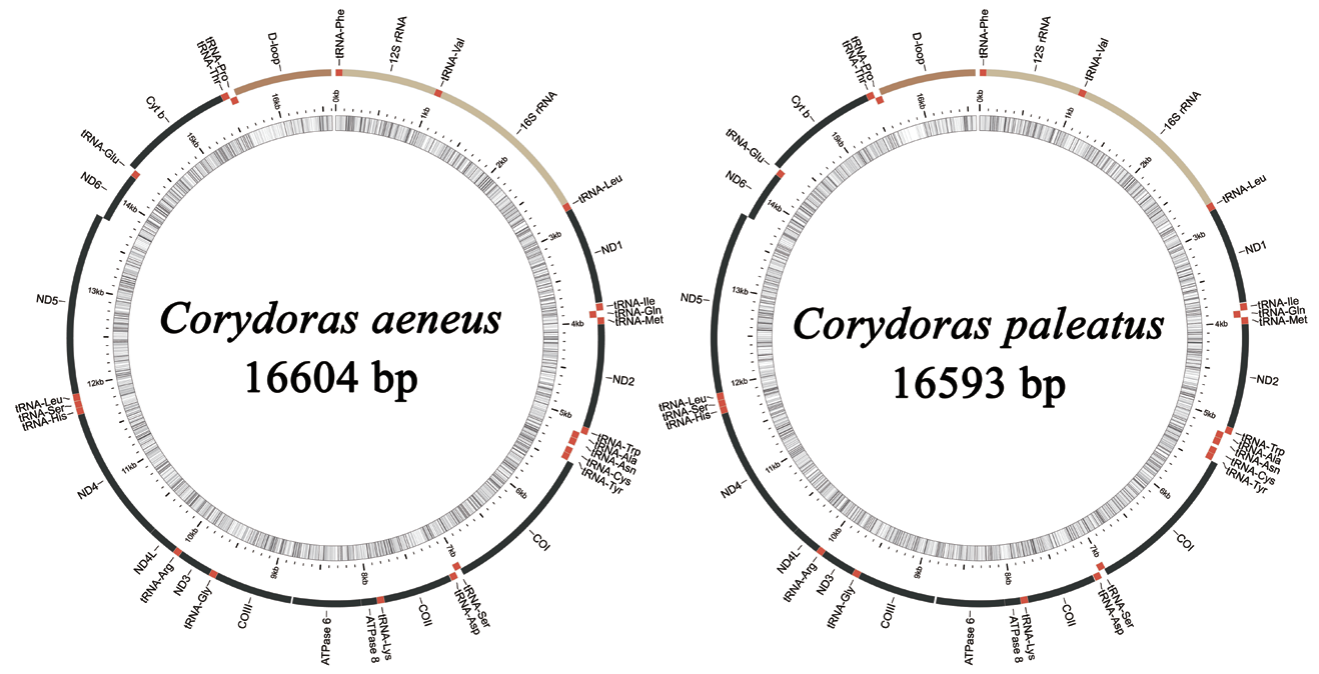
Gene maps of the two newly sequenced *Corydoras* species.

The complete mitogenomes of *C.aeneus* and *C.paleatus* comprising 16,604 and 16,593 bp, respectively, were submitted to GenBank (accession nos. MZ571336 and MZ571337, respectively) (Fig. 1, Table 2). The two mitogenomes were circular and contained 37 mitochondrial genes (13 PCGs, 22 tRNA genes, and two rRNA genes) and one D-loop. The position of each gene in the mitogenome was identical to that in other species of *Corydoras* (Saitoh et al. 2003; Moreira et al. 2016a, 2017; Liu et al. 2019a, b, c, d; Chen et al. 2020; Lv et al. 2020). One of the 13 PCGs (ND6) and eight tRNAs (tRNA-Ala, tRNA-Cys, tRNA-Glu, tRNA-Asn, tRNA-Pro, tRNA-Gln, tRNA-Ser(TGA), and tRNA-Tyr) were encoded by the light chain (-), whereas the other 28 genes, including 12 PCGs, 14 tRNAs, two rRNAs, and one D-loop, were encoded by the heavy chain (+) (Fig. 1, Table 2). The 44 mitogenomes of Siluriformes (Nakatani et al. 2011; Liu et al. 2016; Moreira et al 2016b, 2018; Resende et al. 2016; Magalhães et al. 2017; Parente et al. 2017; Parente et al. 2018; Pereira et al. 2019; Ren et al. 2019; Rocha-Reis et al. 2020; Meng et al. 2021; Zhang et al. 2021) used in this study were compared, and the gene composition and order were consistent (Suppl. material 1: Fig. S1). The nucleotide composition of the two entire mitogenomes was as follows: *C.aeneus* A = 5417 (32.63%), T = 4299 (25.89%), G = 2451 (14.76%), C = 4437 (26.72%) and *C.paleatus* A = 5380 (32.42%), T = 4282 (25.81%), G = 2481 (14.95%), C = 4450 (26.82%). The two mitogenomes (values for *C.aeneus* followed by values for *C.paleatus*) had high A+T contents of 58.52% and 58.23% (Suppl. material 1: Table S2), including 58.08% and 57.67% in PCGs, 56.97% and 57.04% in tRNA genes, 59.70% and 59.10% in 16S rRNA, 55.30% in 12S rRNA, and 67.51% and 68.21% in the D-loop, respectively, which agrees with the typical base bias of fish mitogenomes (Gadaleta et al. 1989; Manchado et al. 2007; Xu et al. 2011). The overall AT and GC skew values in the entire mitogenome of *C.aeneus* were 0.115 and -0.288 and in *C.paleatus* were 0.114 and -0.284, respectively. The GC skew value of the eleven mitogenomes of *Corydoras*, except for tRNA, was slightly negative (-0.014 to -0.288), showing a higher occurrence of C than of G. In contrast, AT skew value, except for the second codon position, was slightly positive (0.028 to 0.379), showing a higher content of A than of T. The K2P genetic distances of the eleven mitogenomes of *Corydoras* were all less than 0.12 (Suppl. material 1: Table S3). *C.nattereri* and *C.sterbai* and *C.nattereri* and *C.trilineatus* showed the largest K2P genetic distances among the eleven species of *Corydoras*.

**Table 2.** Characteristic features of *Corydorasaeneus* and *Corydoraspaleatus* mitogenomes (+ denotes heavy strand; - denotes light strand).

Feature	Position				Length (bp)		Start codons		Stop codons		Anticodon	Strand	Intergenic nucleotides	
	*C.aeneus*		*C.paleatus*		*C.aeneus*	*C.paleatus*	*C.a*	*C.p*	*C.a*	*C.p*				
	From	to	From	to									*C.a*	*C.p*
tRNA-Phe	1	68	1	68	68	68					GAA	+	0	0
12S rRNA	69	1014	69	1013	946	945						+	0	0
tRNA-Val	1015	1086	1014	1085	72	72					TAC	+	0	0
16S rRNA	1087	2757	1086	2753	1671	1668						+	0	0
tRNA-Leu	2758	2832	2754	2828	75	75					TAA	+	0	0
ND1	2833	3804	2829	3800	972	972	ATG	ATG	TAG	TAG		+	8	8
tRNA-Ile	3813	3884	3809	3880	72	72					GAT	+	-2	-2
tRNA-Gln	3883	3953	3879	3949	71	71					TTG	-	-1	-1
tRNA-Met	3953	4022	3949	4018	70	70					CAT	+	0	0
ND2	4023	5067	4019	5063	1045	1045	ATG	ATG	T	T		+	0	0
tRNA-Trp	5068	5139	5064	5134	72	71					TCA	+	1	1
tRNA-Ala	5141	5209	5136	5204	69	69					TGC	-	1	1
tRNA-Asn	5211	5283	5206	5278	73	73					GTT	-	30	31
tRNA-Cys	5314	5380	5310	5377	67	68					GCA	-	-1	-1
tRNA-Tyr	5380	5449	5377	5446	70	70					GTA	-	1	1
COI	5451	7010	5448	7007	1560	1560	GTG	GTG	AGG	AGG		+	-13	-13
tRNA-Ser	6998	7068	6995	7065	71	71					TGA	-	4	4
tRNA-Asp	7073	7141	7070	7138	69	69					GTC	+	4	6
COII	7146	7836	7145	7835	691	691	ATG	ATG	T	T		+	0	0
tRNA-Lys	7837	7910	7836	7909	74	74					TTT	+	1	1
ATPase 8	7912	8079	7911	8078	168	168	ATG	ATG	TAA	TAA		+	-10	-10
ATPase 6	8070	8753	8069	8752	684	684	ATG	ATG	TAA	TAA		+	17	21
COIII	8771	9554	8774	9557	784	784	ATG	ATG	T	T		+	0	0
tRNA-Gly	9555	9626	9558	9629	72	72					TCC	+	0	0
ND3	9627	9975	9630	9978	349	349	ATG	ATG	T	T		+	0	0
tRNA-Arg	9976	10045	9979	10048	70	70					TCG	+	0	0
ND4L	10046	10342	10049	10345	297	297	ATG	ATG	TAA	TAA		+	-7	-7
ND4	10336	11716	10339	11719	1381	1381	ATG	ATG	T	T		+	0	0
tRNA-His	11717	11786	11720	11789	70	70					GTG	+	0	0
tRNA-Ser	11787	11853	11790	11856	67	67					GCT	+	1	1
tRNA-Leu	11855	11927	11858	11930	73	73					TAG	+	0	0
ND5	11928	13754	11931	13757	1827	1827	ATG	ATG	TAA	TAA		+	-4	-4
ND6	13751	14266	13754	14269	516	516	ATG	ATG	TAA	TAA		-	0	0
tRNA-Glu	14267	14335	14270	14337	69	68					TTC	-	2	3
Cyt b	14338	15475	14341	15478	1138	1138	ATG	ATG	T	T		+	0	0
tRNA-Thr	15476	15548	15479	15550	73	72					TGT	+	-2	-2
tRNA-Pro	15547	15616	15549	15618	70	70					TGG	-	0	0
D-loop	15617	16604	15619	16593	988	975							0	0

### ﻿Protein-coding genes

The 13 PCGs of the two new mitogenomes and those of the previously published nine mitogenomes of *Corydoras* contained COI–COIII, ND1–ND6, ND4L, two ATPases, and one Cyt-b, similar to that in other Siluriformes (Nakatani et al. 2011; Liu et al. 2016; Moreira et al. 2016b; Resende et al. 2016; Magalhães et al. 2017; Parente et al. 2017; Moreira 2018; Parente et al. 2018; Pereira et al. 2019; Ren et al. 2019; Rocha-Reis et al. 2020; Meng et al. 2021; Zhang et al. 2021). The total lengths of PCGs in the eleven mitogenomes of *Corydoras* were 11,400–11,414 bp, accounting for 67.84–69.24% of the entire mitogenome. Similar to the mitogenomes of other species of Loricarioidei, ND5 and ATPase 8 were largest (1,827 bp) and smallest (168 bp), respectively. Most PCGs stringently start with an ATG start codon, except for all COIs, which start with GTG, *C.nattereri* COIII (Moreira et al. 2016a) which starts with GCA, and *C.schwartzi* COII (Moreira et al. 2017), which starts with CCA (Suppl. material 1: Table S4). Most PCGs are stringently terminated by the stop codon TAR (TAA/TAG) or an incomplete stop codon T, except for all COIs, which terminate with AGG and *C.schwartzi* ATPase 6 and *C.nattereri* ND3, which terminate with TA. The presence of a truncated stop codon is common among vertebrate mitochondrial genes and is thought to be introduced by posttranscriptional poly-adenylation.

Similar to most previously sequenced members of Loricarioidei, the AT-skews (0.033 to 0.052) and GC-skews (-0.268 to -0.299) of the PCGs were similar among the eleven species of *Corydoras* (Suppl. material 1: Table S2). Summaries of the relative synonymous codon usage and the number of amino acids in the annotated PCGs are presented in Suppl. material 1: Figs S2, S3. The PCGs of the eleven mitogenomes of *Corydoras* (Saitoh et al. 2003; Moreira et al. 2016a, 2017; Liu et al. 2019a, b, c, d; Chen et al. 2020; Lv et al. 2020) translate into 3,798–3,802 codons and showed very similar codon usage, excluding the stop codons (26–28 bp). Ile (310.82 ± 2.69 codons), Thr (312.64 ± 2.27 codons), Ala (312.73 ± 3.08 codons), and Leu1 (CUN) (475.45 ± 12.89 codons) were the four most predominant codon families and may be associated with the coding function of the chondriosome. In contrast, Cys (24.91 ± 0.79 codons) and Ser1 (AGN) (52.18 ± 0.83 codons) had the smallest number of codons. A/T rather than G/C bias was observed in the third position, as almost all frequently used codons ended with A/T. The synonymous codon preferences for the eleven species of *Corydoras* were conserved, possibly because of the close relationships among members of this genus.

**Figure 2.**
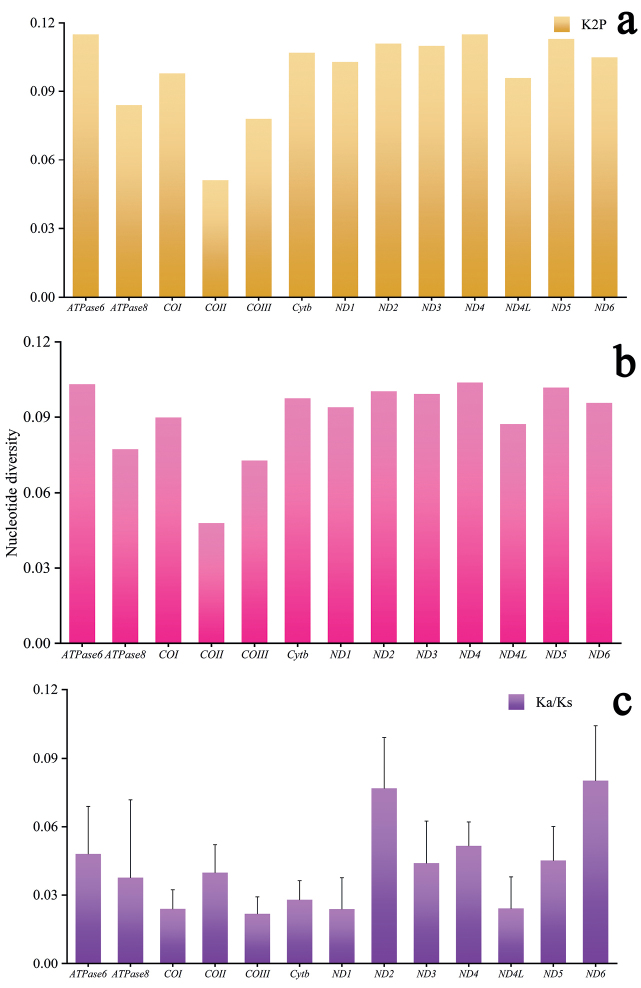
K2P genetic distance **a** nucleotide diversity **b** Ka/Ks ratio **c** analyses of protein-coding genes among the eleven *Corydoras* mitogenomes.

To reveal the evolutionary pattern of the PCGs, the Ka/Ks, nucleotide diversity, and K2P genetic distance across all mitogenomes of *Corydoras* were calculated for each aligned PCG. The K2P genetic distances of 13 PCGs were all less than 0.12 (Fig. 2a). Among the PCGs detected, ND4 and ATPase 8 showed the largest K2P genetic distance among the eleven species of *Corydoras*, followed by ND2 and ND3. The nucleotide diversity of the 13 PCGs was less than 0.11 (Fig. 2b). ND4 showed the highest nucleotide diversity, whereas COII showed the lowest diversity. To investigate the selective pressure across species of *Corydoras*, the Ka/Ks ratios of the PCGs of each mitogenome were estimated (Fig. 2c). The Ka/Ks value was highest for ND6, followed by ND2; the lowest values were observed for COI, COIII, ND1, and ND4L. All 13 PCGs showed Ka/Ks << 1, suggesting that all PCGs of *Corydoras* evolved under purifying selection.

### ﻿tRNAs, ribosomal RNAs, and control region

The total lengths of the 22 tRNA genes ranged from 1,438 (*C.schwartzi*) to 1,561 bp (*C.arcuatus* and *C.panda*), whereas individual tRNA genes typically ranged from 58 to 75 bp. All tRNA genes displayed the expected cloverleaf secondary structures with normal base pairing, except for tRNA-Ser(GCT), which lacked the DHU stem (Suppl. material 1: Fig. S4), forming a loop commonly found in other vertebrates (Ojala et al. 1981; Gadaleta et al. 1989; Wolstenholme 1992). The A+T contents of these tRNAs were 56.55–57.58%. All AT-skew and GC-skew values were slightly positive, indicating a slight bias toward the use of A and G in the tRNAs (Suppl. material 1: Table S2). These rRNA genes are between tRNA-Phe and tRNA-Leu(TAA) and are separated by tRNA-Val. The average total size of the two rRNAs was 2,614 bp, and the average A+T content was 57.89%. Like the tRNAs, all AT-skew values were positive, whereas all GC-skew values were negative, indicating that rRNAs favor C compared to tRNAs in *Corydoras*.

The control region (D-loop), also known as the A+T rich region that contains hypervariable non-coding sequences and regulates the replication and transcription of mitochondrial DNA, is the largest non-coding region and is located between tRNA-Pro and tRNA-Phe in these mitogenomes. Compared with PCGs, the D-loop displayed a higher mutation rate and the highest variation throughout the mitogenome; thus, this region is dominant and can be used to evaluate intraspecies variations. The D-loops in the eleven species of *Corydoras* were 718‒1,218 bp. Compared with the other four regions (entire genome, PCGs, tRNAs, and rRNAs), the control region showed the highest A+T content, ranging from 66.77% to 71.87%. Like the rRNAs, all AT-skew values were positive, and all GC-skew values were negative.

### ﻿Phylogenetic analysis

**Figure 3.**
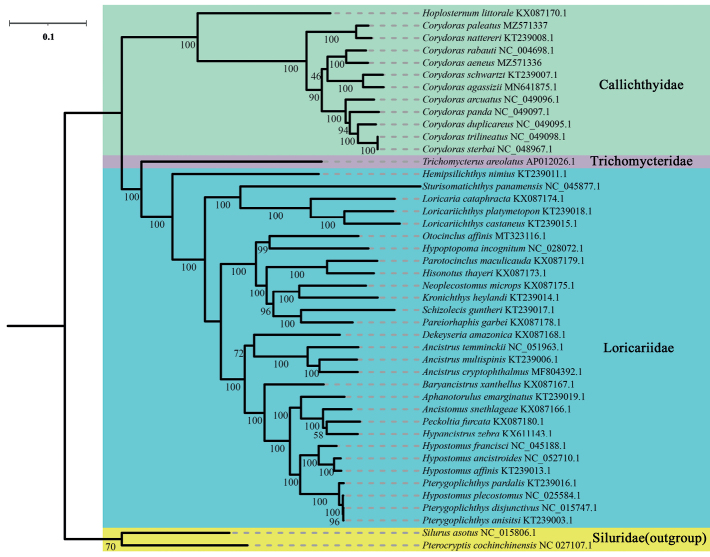
Phylogenetic trees of 44 Siluriformes species using concatenated nucleotide sequences of 13 protein-coding genes and two rRNAs using the maximum likelihood method. Numbers in the ML tree represent SH-aLRT support/ultrafast bootstrap support values.

**Figure 4.**
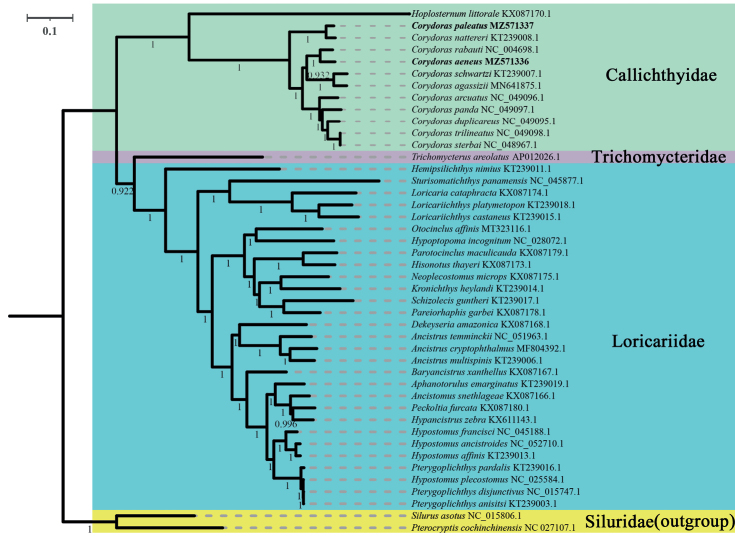
Phylogenetic tree of 44 Siluriformes species using concatenated nucleotide sequences of 13 protein-coding genes and two rRNAs via the Bayesian interference method. Applicable posterior probability values are shown.

To determine the phylogenetic relationships within the suborder Loricarioidei and family Callichthyidae, we obtained the concatenated nucleotide sequences of 13 PCGs and two rRNAs from 42 species of Loricarioidei. Phylogenetic analyses based on both ML and BI methods revealed same topologies, which also generally agreed with those presented in previous studies (Alexandrou et al. 2011; Lujan et al. 2015; Moreira et al. 2017; Roxo et al. 2019) (Figs 3, 4). These analyses confirmed that the genus *Corydoras* was part of the monophyletic family Callichthyidae.

Both Callichthyidae and Loricariidae were recovered as monophyletic with very high support values (BI posterior probabilities, PP = 1; ML bootstrap, BS = 100). The 44 species of Siluriformes were divided into four major clades corresponding to the families SiluridaeCallichthyidae, Trichomycteridae, and Loricariidae. The target species *C.aeneus* and *C.paleatus* were clustered into two clades (*C.aeneus* + *C.rabauti*) and (*C.paleatus* + *C.nattereri*) with a high nodal support value (PP = 1; BS = 100). The eleven species of the genus *Corydoras* clustered together quite well [((*C.aeneus* + *C.rabauti*) + (*C.schwartzi* + *C.agassizii*)) + (*C.arcuatus* + (*C.panda* + (*C.duplicareus* + (*C.sterbai* + *C.trilineatus*))))] + [(*C.paleatus* + *C.nattereri*)]. *Corydorastrilineatus* and *C.sterbai* have short, almost non-existent branch lengths; thus, they are likely the same species. The K2P genetic distances of these two species are 0.000 (Suppl. material 1: Table S3), which verifies that they are the same species. This may be caused by incorrect identification, taxonomic problems (these two species are, in fact, synonymous), and/or introgressive hybridization. Moreover, in the family Loricariidae, the genera *Ancistrus* and *Loricariichthys* were clustered into monophyletic clades [(*A. cryptophthalmus + A.multispinis*) + *A.temminckii*] and (*L.castaneus + L.platymetopon*) with a high nodal support value (PP = 1; BS = 100). There was a paraphyletic relationship between the genera *Hypostomus* and *Pterygoplichthys*, [*H.francisci* + (*H.ancistroides* + *H.affinis*), *P.pardalis* + (*H.plecostomus* + (*P.anisitsi* + *P.disjunctivus*))]. Our results demonstrate that the concatenated nucleotide sequences of the 13 PCGs and two rRNAs were useful for determining the phylogenetic relationships of the order Siluriformes. These results can be used to improve classification of the families Callichthyidae and Loricariidae.

## ﻿Conclusions

Using next-generation sequencing methods, the complete mitogenomes of the bronze *C.aeneus* and peppered *C.paleatus* were analyzed and compared with those of nine members of *Corydoras*. The complete mitogenomes of *C.aeneus* and *C.paleatus* comprised 16,604 and 16,593 bp, respectively. The two mitogenomes had high A+T contents (58.52% in *C.aeneus* and 58.23% in *C.paleatus*), a phenomenon that agrees with the typical base bias of ichthyic mitogenomes. Our results indicate that the mitogenome features, including genome size, gene content, and gene arrangement, in *Corydoras* are highly conserved. Phylogenetic analysis was performed with 42 species of Loricarioidei and two outgroup species. These analyses confirmed the occurrence of the genus *Corydoras* within the monophyletic family Callichthyidae. The complete mitogenome information, including the gene content, gene orders, genome structure, base compositions, evolutionary features, codon usage, gene arrangement, and phylogenetic analyses, provides a basis for future studies on the population genetic and evolution of *Corydoras* and related groups.

## Supplementary materials

Supplementary material 1 COI sequences of *Corydorasaeneus* and *C.paleatus* Tables S1–S4, Figs S1–S4 Data type docx file Explanation note COI sequences of *Corydorasaeneus* and *C.paleatus*. Table S1. Best substitution models for Bayesian inference (BI) and maximum-likelihood (ML) analyses. Table S2. Summarized mitogenomic characteristics of the eleven *Corydoras* species investigated in this study. Table S3. The K2P genetic distances of the eleven mitogenomes of *Corydoras*. Table S4. Start nd stop codons of protein-coding genes in the eleven *Corydoras* mitogenomes. Figure S1. Gene orders of mitogenomes of the studied species. Figure S2. Relative synonymous codon usage of 13 protein-coding genes in the mitogenomes of eleven *Corydoras* species. Figure S3. Codon usage patterns of eleven *Corydoras* mitogenomes. Figure S4. Secondary structures of tRNA-Ser(GCT) in the two newly sequenced *Corydoras* species. https://binary.pensoft.net/file/637451This dataset is made available under the Open Database License (http://opendatacommons.org/licenses/odbl/1.0/). The Open Database License (ODbL) is a license agreement intended to allow users to freely share, modify, and use this Dataset while maintaining this same freedom for others, provided that the original source and author(s) are credited.Cheng-He Sun, Qi Huang, Xiao-Shu Zeng, Sha Li, Xiao-Li Zhang, Ya-Nan Zhang, Jian Liao, Chang-Hu Lu, Bo-Ping Han, Qun Zhang
