# Supplementary material for: ﻿Comparative analysis of the mitogenomes of two Corydoras (Siluriformes, Loricarioidei) with nine known Corydoras, and a phylogenetic analysis of Loricarioidei
Source: Zookeys. 2022 Jan 24;1083:89–107. doi: 10.3897/zookeys.1083.76887 (PMC8803743; doi:10.3897/zookeys.1083.76887)
Supplement: Supplementary material 1 — COI sequences of Corydorasaeneus and C.paleatus Tables S1–S4, Figs S1–S4 [file zookeys-1083-089-s001.docx]

**Supplementary material**

>COI sequence of *Corydoras aeneus*

CGTGCTCAACCTTTTGGTGCCTGAGCTGGTATAGTCGGAACCGCCTTAAGCCTACTAATTCGAGCTGAGCTAAATCAACCAGGCTCCCTTCTGGGCGACGATCAAATTTACAATGTTATCGTTACGGCGCACGCCTTCATCATAATCTTCTTTATAGTAATGCCAATTATAATTGGTGGTTTCGGAAATTGACTCATCCCTCTAATAATTGGAGCACCAGATATAGCATTTCCACGAATAAACAATATGAGCTTCTGACTACTTCCACCCTCATTCCTCCTTCTACTAGCATCCTCTGGAGTAGAAGCAGGAGCAGGAACAGGCTGAACTGTTTACCCACCGCTCGCAGGAAATCTTGCACACGCAGGAGCTTCTGTTGACTTAACAATTTTCTCTCTCCATCTCGCTGGTGTTTCTTCTATTTTAGGAGCAATCAACTTCATTACAACAATTATTAACATGAAGCCCCCTGCTATTTCACAATATCAAACACCACTATTCGTATGAGCAGTATTAATTACCGCTGTTCTACTTCTTCTGTCCCTTCCAGTCCTAGCCGCTGGAATTACTATACTACTCACAGACCGTAATCTAAACACCACCTTCTTTGACCCCGCAGGAGGAGGAGACCCCATCCTATACCAGCATTTATTCTGATCCTTCGC

>COI sequence of *Corydoras paleatus*

ACCGCCTTAAGCCTACTAATTCGAGCAGAGCTAAATCAACCTGGCTCCCTTCTAGGCGACGATCAAATTTATAACGTTATCGTTACGGCGCACGCCTTCATTATAATCTTCTTTATAGTAATGCCAATTATGATCGGCGGCTTTGGAAACTGACTTGTTCCCCTAATAATTGGGGCACCAGATATGGCATTTCCACGAATAAATAACATAAGCTTCTGACTACTCCCACCCTCATTCCTCCTTCTTCTAGCATCCTCAGGGGTAGAAGCAGGTGCAGGAACAGGCTGAACTGTTTACCCACCACTTGCAGGAAACCTTGCCCATGCAGGGGCCTCTGTTGACTTAACAATTTTTTCCCTCCACCTTGCAGGTGTCTCCTCCATTCTAGGGGCAATCAATTTCATCACAACAATCATTAACATAAAACCTCCTGCAATCTCACAATATCAAACACCACTATTCGTGTGGGCAGTACTAATTACCGCTGTCCTACTGCTCCTGTCCCTTCCAGTCCTAGCCGCTGGAATCACTATATTACTTACAGACCGTAACCTAAATACTACTTTCTTTGACCCGGCGGGAGGAGGAGACCCAATTCTTTACCAACACTTATTCTGATT

Table S1. Best substitution models for Bayesian inference (BI) and maximum-likelihood (ML) analyses.

|  | ML | BI |
| --- | --- | --- |
| ATPase 6 | GTR+F+R4 | GTR+F+I+G4 |
| ATPase 8 | TPM3u+F+I+G4 | HKY+F+I+G4 |
| COI | TIM2+F+I+G4 | GTR+F+I+G4 |
| COII | TIM2+F+I+G4 | GTR+F+I+G4 |
| COIII | TIM2+F+I+G4 | GTR+F+I+G4 |
| Cyt b | GTR+F+R4 | GTR+F+I+G4 |
| ND1 | GTR+F+I+G4 | GTR+F+I+G4 |
| ND2 | GTR+F+I+G4 | GTR+F+I+G4 |
| ND3 | GTR+F+R4 | GTR+F+I+G4 |
| ND4 | GTR+F+R4 | GTR+F+I+G4 |
| ND4L | TIM2+F+I+G4 | GTR+F+I+G4 |
| ND5 | GTR+F+R4 | GTR+F+I+G4 |
| ND6 | K3Pu+F+I+G4 | HKY+F+I+G4 |
| 12S rRNA | GTR+F+R4 | GTR+F+I+G4 |
| 16S rRNA | GTR+F+R4 | GTR+F+I+G4 |

Table S2. Summarized mitogenomic characteristics of the eleven *Corydoras* species investigated in this study.

|  | Species | Whole genome | Protein-coding genes | 1st codon position | 2nd codon position | 3rd codon position | tRNA genes | *16S rRNA* | *12S rRNA* | Control region |
| --- | --- | --- | --- | --- | --- | --- | --- | --- | --- | --- |
| A+T Contents  (%) | *C. aeneus* | 58.52 | 58.08 | 50.08 | 59.76 | 64.33 | 56.97 | 59.70 | 55.30 | 67.51 |
|  | *C. agassizii* | 58.48 | 58.13 | 49.71 | 59.81 | 64.81 | 57.50 | 59.30 | 55.00 | 66.77 |
|  | *C. arcuatus* | 58.51 | 57.70 | 50.55 | 59.65 | 62.84 | 57.14 | 59.20 | 55.00 | 69.64 |
|  | *C. duplicareus* | 59.45 | 59.02 | 50.76 | 59.81 | 66.41 | 57.34 | 60.00 | 55.60 | 69.90 |
|  | *C. nattereri* | 57.88 | 57.30 | 49.92 | 59.69 | 62.22 | 56.55 | 59.00 | 54.80 | 68.51 |
|  | *C. paleatus* | 58.23 | 57.67 | 49.95 | 59.76 | 63.23 | 57.04 | 59.10 | 55.30 | 68.21 |
|  | *C. panda* | 58.82 | 58.45 | 50.58 | 59.73 | 64.97 | 57.14 | 59.30 | 55.10 | 68.71 |
|  | *C. rabauti* | 58.64 | 57.89 | 50.01 | 59.85 | 63.74 | 57.34 | 59.70 | 55.00 | 68.97 |
|  | *C. schwartzi* | 58.66 | 58.17 | 49.87 | 59.66 | 64.90 | 57.58 | 59.20 | 54.80 | 71.87 |
|  | *C. sterbai* | 59.12 | 58.65 | 50.63 | 59.81 | 65.44 | 57.14 | 59.70 | 55.40 | 69.89 |
|  | *C. trilineatus* | 58.94 | 58.65 | 50.63 | 59.81 | 65.44 | 57.12 | 59.70 | 55.40 | 67.91 |
| AT-skew | *C. aeneus* | 0.115 | 0.048 | 0.128 | -0.372 | 0.379 | 0.035 | 0.257 | 0.243 | 0.031 |
|  | *C. agassizii* | 0.116 | 0.048 | 0.134 | -0.373 | 0.372 | 0.050 | 0.255 | 0.237 | 0.043 |
|  | *C. arcuatus* | 0.106 | 0.034 | 0.117 | -0.372 | 0.355 | 0.049 | 0.257 | 0.242 | 0.052 |
|  | *C. duplicareus* | 0.107 | 0.037 | 0.117 | -0.374 | 0.348 | 0.027 | 0.251 | 0.244 | 0.052 |
|  | *C. nattereri* | 0.114 | 0.046 | 0.136 | -0.371 | 0.377 | 0.028 | 0.241 | 0.259 | 0.039 |
|  | *C. paleatus* | 0.114 | 0.048 | 0.133 | -0.369 | 0.378 | 0.028 | 0.243 | 0.250 | 0.050 |
|  | *C. panda* | 0.106 | 0.035 | 0.114 | -0.371 | 0.349 | 0.045 | 0.261 | 0.241 | 0.072 |
|  | *C. rabauti* | 0.113 | 0.046 | 0.124 | -0.371 | 0.379 | 0.047 | 0.257 | 0.240 | 0.050 |
|  | *C. schwartzi* | 0.122 | 0.052 | 0.137 | -0.374 | 0.381 | 0.041 | 0.263 | 0.245 | 0.062 |
|  | *C. sterbai* | 0.105 | 0.033 | 0.122 | -0.373 | 0.338 | 0.036 | 0.257 | 0.249 | 0.066 |
|  | *C. trilineatus* | 0.106 | 0.033 | 0.122 | -0.373 | 0.338 | 0.035 | 0.257 | 0.249 | 0.068 |
| GC-skew | *C. aeneus* | -0.288 | -0.312 | -0.014 | -0.328 | -0.712 | 0.060 | -0.070 | -0.106 | -0.159 |
|  | *C. agassizii* | -0.290 | -0.311 | -0.021 | -0.330 | -0.706 | 0.044 | -0.078 | -0.099 | -0.146 |
|  | *C. arcuatus* | -0.268 | -0.291 | -0.003 | -0.331 | -0.631 | 0.034 | -0.074 | -0.101 | -0.112 |
|  | *C. duplicareus* | -0.279 | -0.304 | -0.004 | -0.329 | -0.715 | 0.065 | -0.069 | -0.107 | -0.136 |
|  | *C. nattereri* | -0.285 | -0.299 | -0.019 | -0.329 | -0.638 | 0.053 | -0.085 | -0.096 | -0.201 |
|  | *C. paleatus* | -0.284 | -0.304 | -0.018 | -0.328 | -0.665 | 0.063 | -0.081 | -0.090 | -0.181 |
|  | *C. panda* | -0.269 | -0.294 | -0.001 | -0.331 | -0.667 | 0.046 | -0.071 | -0.096 | -0.164 |
|  | *C. rabauti* | -0.287 | -0.311 | -0.013 | -0.333 | -0.695 | 0.041 | -0.067 | -0.101 | -0.164 |
|  | *C. schwartzi* | -0.299 | -0.319 | -0.022 | -0.329 | -0.733 | 0.056 | -0.091 | -0.115 | -0.099 |
|  | *C. sterbai* | -0.273 | -0.297 | -0.009 | -0.330 | -0.671 | 0.055 | -0.071 | -0.107 | -0.130 |
|  | *C. trilineatus* | -0.274 | -0.297 | -0.009 | -0.330 | -0.671 | 0.055 | -0.071 | -0.107 | -0.133 |

Table S3. The K2P genetic distances of the eleven mitogenomes of *Corydoras.*

| No. |  | 1 | 2 | 3 | 4 | 5 | 6 | 7 | 8 | 9 | 10 |
| --- | --- | --- | --- | --- | --- | --- | --- | --- | --- | --- | --- |
| 1 | *C. aeneus* |  |  |  |  |  |  |  |  |  |  |
| 2 | *C. agassizii* | 0.086 |  |  |  |  |  |  |  |  |  |
| 3 | *C. arcuatus* | 0.086 | 0.093 |  |  |  |  |  |  |  |  |
| 4 | *C. duplicareus* | 0.082 | 0.094 | 0.056 |  |  |  |  |  |  |  |
| 5 | *C. nattereri* | 0.104 | 0.108 | 0.107 | 0.106 |  |  |  |  |  |  |
| 6 | *C. paleatus* | 0.102 | 0.106 | 0.106 | 0.105 | 0.029 |  |  |  |  |  |
| 7 | *C. panda* | 0.084 | 0.098 | 0.061 | 0.054 | 0.108 | 0.107 |  |  |  |  |
| 8 | *C. rabauti* | 0.044 | 0.084 | 0.084 | 0.079 | 0.102 | 0.100 | 0.084 |  |  |  |
| 9 | *C. schwartzi* | 0.084 | 0.043 | 0.094 | 0.091 | 0.108 | 0.106 | 0.096 | 0.083 |  |  |
| 10 | *C. sterbai* | 0.085 | 0.098 | 0.059 | 0.040 | **0.110** | 0.109 | 0.055 | 0.083 | 0.094 |  |
| 11 | *C. trilineatus* | 0.085 | 0.098 | 0.059 | 0.040 | **0.110** | 0.109 | 0.055 | 0.083 | 0.094 | 0.000 |

Table S4. Start and stop codons of protein-coding genes in the eleven *Corydoras* mitogenomes.

|  | Feature | *ATPase 6* | *ATPase 8* | *COI* | *COII* | *COIII* | *Cyt b* | *ND1* | *ND2* | *ND3* | *ND4* | *ND4L* | *ND5* | *ND6* |
| --- | --- | --- | --- | --- | --- | --- | --- | --- | --- | --- | --- | --- | --- | --- |
| Start codons | *C. aeneus* | ATG | ATG | GTG | ATG | ATG | ATG | ATG | ATG | ATG | ATG | ATG | ATG | ATG |
|  | *C. agassizii* | ATG | ATG | GTG | ATG | ATG | ATG | ATG | ATG | ATG | ATG | ATG | ATG | ATG |
|  | *C. arcuatus* | ATG | ATG | GTG | ATG | ATG | ATG | ATG | ATG | ATG | ATG | ATG | ATG | ATG |
|  | *C. duplicareus* | ATG | ATG | GTG | ATG | ATG | ATG | ATG | ATG | ATG | ATG | ATG | ATG | ATG |
|  | *C. nattereri* | ATG | ATG | GTG | ATG | **GCA** | ATG | ATG | ATG | ATG | ATG | ATG | ATG | ATG |
|  | *C. paleatus* | ATG | ATG | GTG | ATG | ATG | ATG | ATG | ATG | ATG | ATG | ATG | ATG | ATG |
|  | *C. panda* | ATG | ATG | GTG | ATG | ATG | ATG | ATG | ATG | ATG | ATG | ATG | ATG | ATG |
|  | *C. rabauti* | ATG | ATG | GTG | ATG | ATG | ATG | ATG | ATG | ATG | ATG | ATG | ATG | ATG |
|  | *C. schwartzi* | ATG | ATG | GTG | **CCA** | ATG | ATG | ATG | ATG | ATG | ATG | ATG | ATG | ATG |
|  | *C. sterbai* | ATG | ATG | GTG | ATG | ATG | ATG | ATG | ATG | ATG | ATG | ATG | ATG | ATG |
|  | *C. trilineatus* | ATG | ATG | GTG | ATG | ATG | ATG | ATG | ATG | ATG | ATG | ATG | ATG | ATG |
| Stop codons | *C. aeneus* | TAA | TAA | AGG | T | T | T | TAG | T | T | T | TAA | TAA | TAA |
|  | *C. agassizii* | TAA | TAA | AGG | T | T | T | TAG | T | T | T | TAA | TAA | TAG |
|  | *C. arcuatus* | TAA | TAA | AGG | T | T | T | TAG | T | T | T | TAA | TAA | TAA |
|  | *C. duplicareus* | TAA | TAA | AGG | T | T | T | TAG | T | T | T | TAA | TAA | TAA |
|  | *C. nattereri* | TAA | TAA | AGG | T | T | T | TAG | **TA** | T | T | TAA | TAA | TAA |
|  | *C. paleatus* | TAA | TAA | AGG | T | T | T | TAG | T | T | T | TAA | TAA | TAA |
|  | *C. panda* | TAA | TAA | AGG | T | T | T | TAG | T | T | T | TAA | TAG | TAA |
|  | *C. rabauti* | TAA | TAA | AGG | T | T | T | TAG | TAG | T | T | TAA | TAA | TAA |
|  | *C. schwartzi* | **TA** | TAA | AGG | T | T | T | TAG | T | T | T | TAA | TAA | TAG |
|  | *C. sterbai* | TAA | TAA | AGG | T | T | T | TAG | T | T | T | TAA | TAA | TAG |
|  | *C. trilineatus* | TAA | TAA | AGG | T | T | T | TAG | T | T | T | TAA | TAA | TAG |


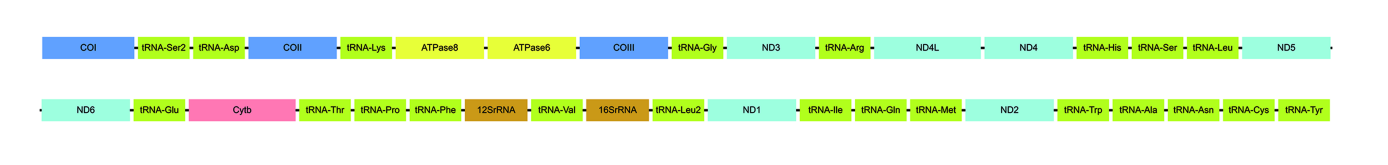


Figure S1. Gene orders of mitogenomes of the studied species.


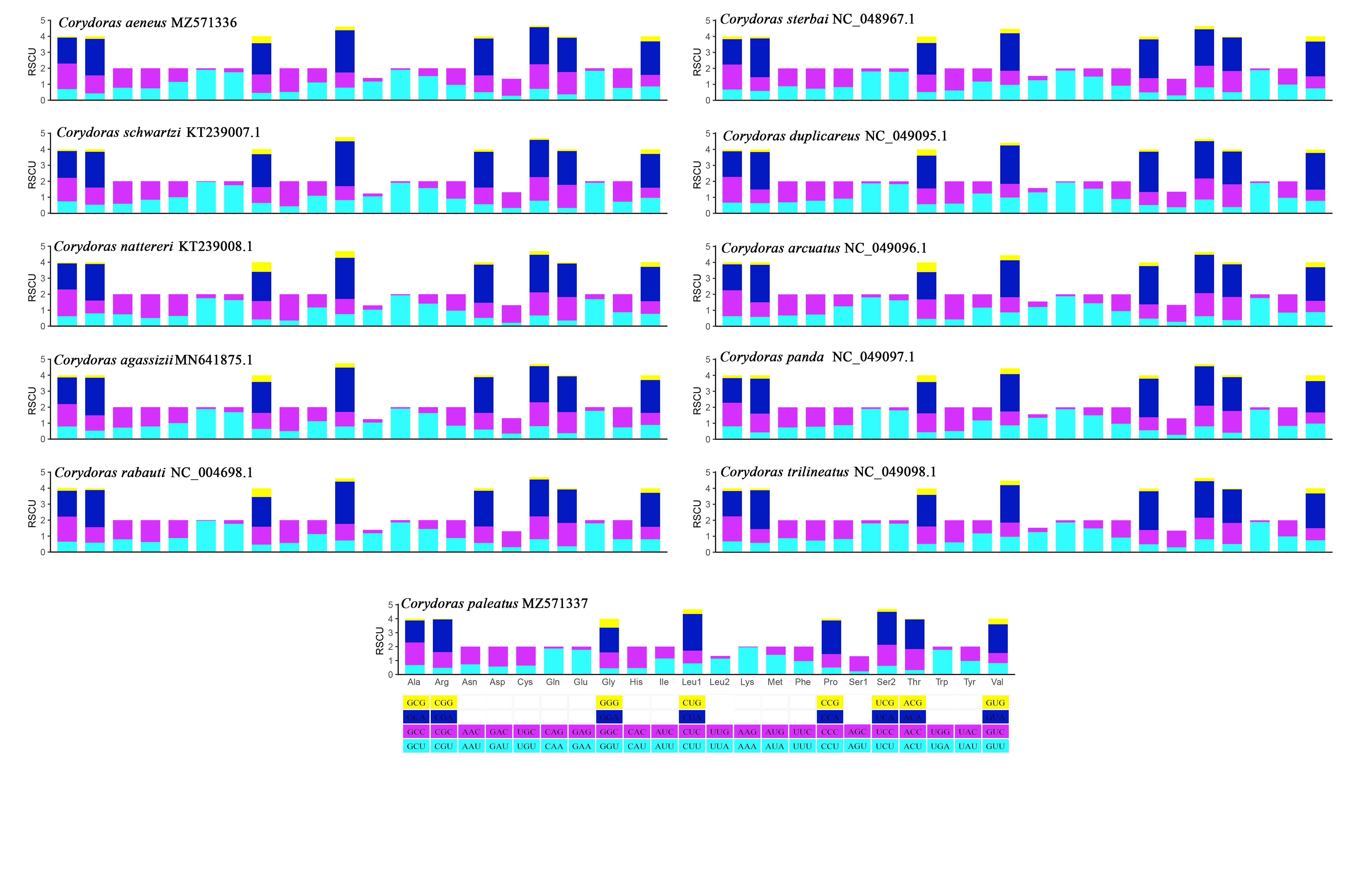


Figure S2. Relative synonymous codon usage of 13 protein-coding genes in the mitogenomes of eleven *Corydoras* species.





Figure S3. Codon usage patterns of eleven *Corydoras* mitogenomes.


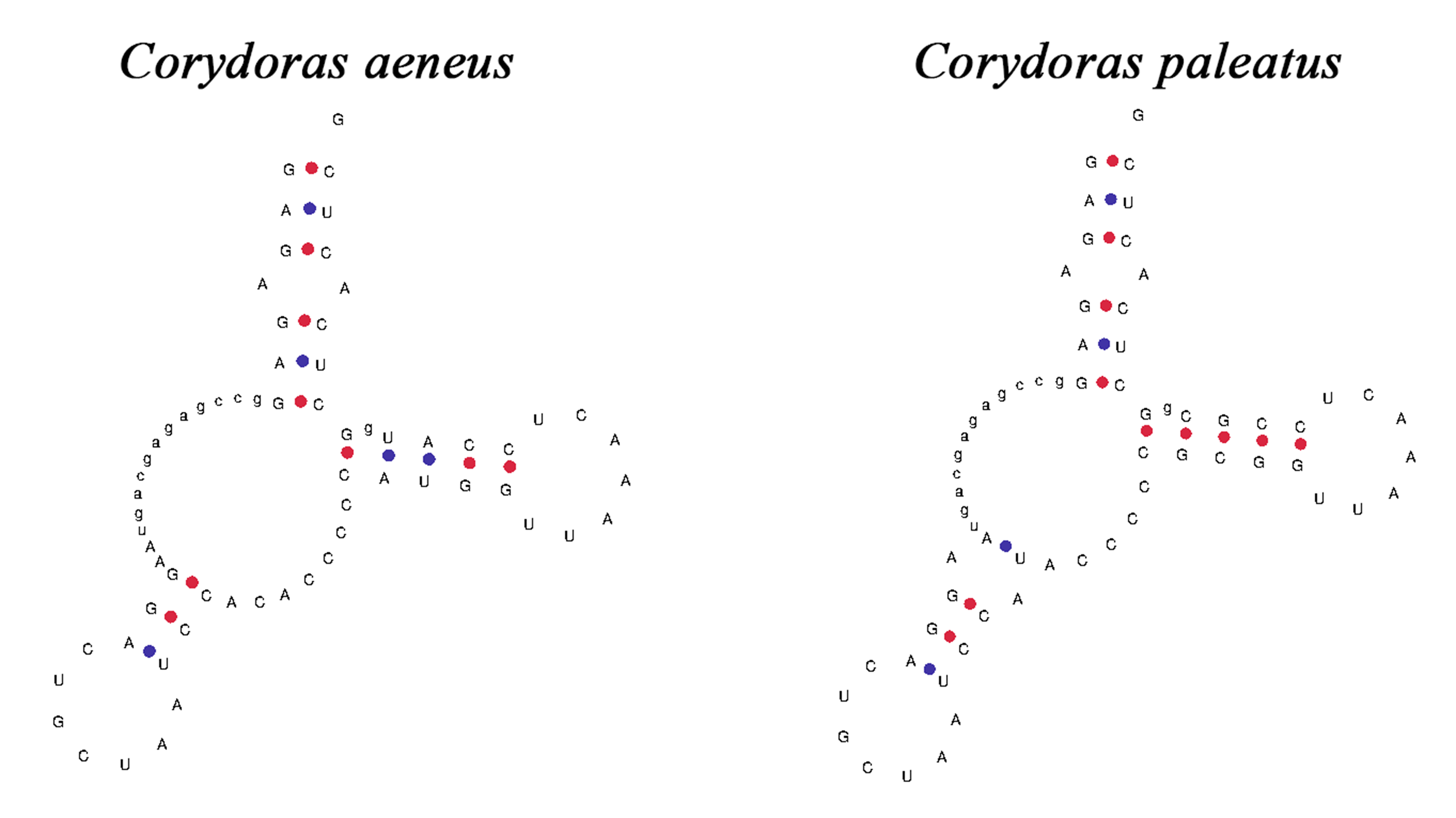


Figure S4. Secondary structures of tRNA-Ser(GCT) in the two newly sequenced *Corydoras* species.
